# *catena*-Poly[[diaquacadmium(II)]bis­(μ-pyridine-3-sulfonato)-κ^2^ *N*:*O*;κ^2^ *O*:*N*]

**DOI:** 10.1107/S1600536808007770

**Published:** 2008-03-29

**Authors:** Zhi-Hui Qiu, Fu-Pei Liang, Qing-Feng Ruan, Shan-Rong Zhao

**Affiliations:** a College of Chemistry and Chemical Engineering, Guangxi Normal University, Guilin 541004, People’s Republic of China; b Faculty of Earth Sciences, China University of Geosciences, Wuhan 430074, People’s Republic of China; c Department of Resources and Environmental Engineering, Guilin University of Technology, Guilin 541004, People’s Republic of China

## Abstract

In the title polymeric complex, [Cd(C_5_H_4_NO_3_S)_2_(H_2_O)_2_]_*n*_, the Cd atom is located on a centre of inversion and is coordinated by two O atoms and two N atoms, derived from four different pyridine-3-sulfonate ligands, and two O atoms derived from two water mol­ecules, forming a distorted *trans*-N_2_O_4_ octa­hedral geometry. The topology of the polymer is a one-dimensional chain mediated by bridging pyridine-3-sulfonate anions. These are connected into a three-dimensional architecture *via* hydrogen bonds.

## Related literature

For related literature, see: Allen (2002 ▶). For related structures, see: Brodersen *et al.* (1980 ▶); Chandrasekhar (1977 ▶); Cotton *et al.* (1992*a* ▶,*b* ▶); van der Lee & Barboiu (2004 ▶); Mäkinen *et al.* (2001 ▶); Walsh & Hathaway (1980 ▶).
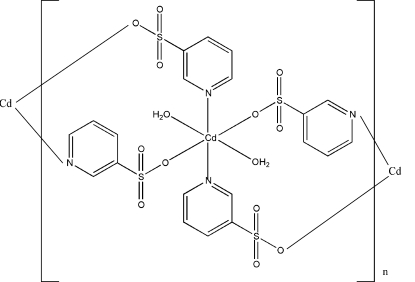


## Experimental

### 

#### Crystal data

[Cd(C_5_H_4_NO_3_S)_2_(H_2_O)_2_]
*M* *_r_* = 464.74
Monoclinic, 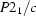

*a* = 7.7480 (11) Å
*b* = 13.264 (2) Å
*c* = 7.3291 (11) Å
β = 97.081 (2)°
*V* = 747.47 (19) Å^3^
*Z* = 2
Mo *K*α radiation
μ = 1.78 mm^−1^
*T* = 294 (2) K
0.26 × 0.22 × 0.18 mm

#### Data collection

Bruker SMART CCD area-detector diffractometer
Absorption correction: multi-scan (*SADABS*; Bruker, 1998 ▶) *T*_min_ = 0.674, *T*_max_ = 0.740
4111 measured reflections
1520 independent reflections
1396 reflections with *I* > 2σ(*I*)
*R*_int_ = 0.023

#### Refinement

*R*[*F* ^2^ > 2σ(*F* ^2^)] = 0.021
*wR*(*F* ^2^) = 0.053
*S* = 1.10
1520 reflections
115 parameters
H atoms treated by a mixture of independent and constrained refinement
Δρ_max_ = 0.42 e Å^−3^
Δρ_min_ = −0.73 e Å^−3^

### 

Data collection: *SMART* (Bruker, 1998 ▶); cell refinement: *SAINT* (Bruker, 1998 ▶); data reduction: *SAINT*; program(s) used to solve structure: *SHELXS97* (Sheldrick, 2008 ▶); program(s) used to refine structure: *SHELXL97* (Sheldrick, 2008 ▶); molecular graphics: *SHELXTL* (Sheldrick, 2008 ▶); software used to prepare material for publication: *SHELXTL*.

## Supplementary Material

Crystal structure: contains datablocks I. DOI: 10.1107/S1600536808007770/tk2252sup1.cif

Structure factors: contains datablocks I. DOI: 10.1107/S1600536808007770/tk2252Isup2.hkl

Additional supplementary materials:  crystallographic information; 3D view; checkCIF report

## Figures and Tables

**Table 1:** Hydrogen-bond geometry (Å, °)

*D*—H⋯*A*	*D*—H	H⋯*A*	*D*⋯*A*	*D*—H⋯*A*
C5—H5⋯O3^i^	0.93	2.39	3.256 (3)	155
C4—H4⋯O2^ii^	0.93	2.55	3.422 (3)	157
O4—H4*B*⋯O3^iii^	0.79 (3)	1.99 (4)	2.780 (3)	176 (3)
O4—H4*A*⋯O2^iv^	0.81 (3)	1.98 (3)	2.773 (3)	168 (3)

Symmetry codes: (i) 
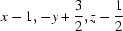
; (ii) 
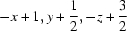
; (iii) 
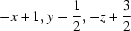
; (iv) 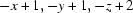.

**Fig. 1.**
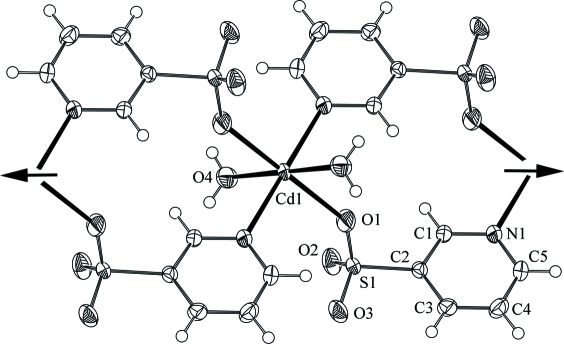
Extended structure in (I) showing the coordination geometry for the Cd atom, the atom labelling scheme and displacement ellipsoids at the 50% probability level. The Cd atom is located at a center of inversion.

**Fig. 2.**
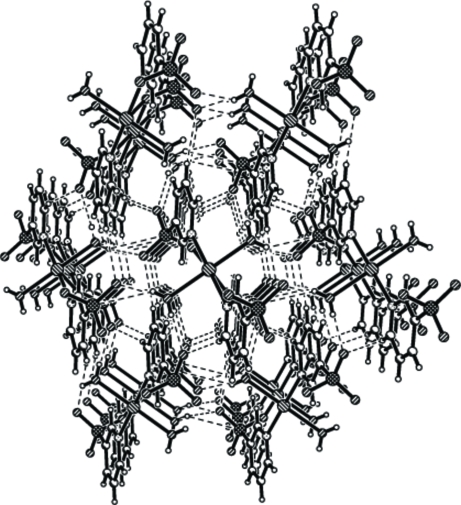
Crystal packing of (I) viewed approximately down the a-direction showing the hydrogen bonding interactions as dashed lines.

## supplementary crystallographic information

### Comment

Complexes or salts based on pyridinesulfonate are very rare in the Cambridge Structural Database (CSD; Version 5.25; Allen, 2002). A six-coordinate complex with pyridine-3-sulfonate ligands that is closely related to the title complex, (I), has been reported (Walsh & Hathaway, 1980). Other pyridine-3-sulfonate complexes are also available (Brodersen *et al.*, 1980; Cotton *et al.*, 1992*a*, b; Mäkinen *et al.*, 2001; van der Lee & Barboiu, 2004), as well as that of the acid (Chandrasekhar, 1977).

In (I), Fig. 1, the Cd atom is located on a centre of inversion and is six-coordinated by two N atoms and two O atoms derived from four different pyridine-3-sulfonate molecules, and two O atoms derived from two water molecules. The resulting *trans*-N_2_O_4_ donor sets defines a distorted octahedral environment for Cd with angles ranging from 84.76 (7) to 180°, Cd—O distances in the range 2.2872 (18) to 2.3113 (17) Å, and Cd—N distances of 2.3233 (18) and 2.3234 (18) Å.

The molecules aggregate *via* bridging pyridine-3-sulfonate anions to form a chain. In the crystal structure, chains are linked into a 3-D architecture *via* hydrogen bonding interactions, Table 1 & Fig. 2.

### Experimental

Pyridine-3-sulfonate, (1 mmol, 159 mg) was dissolved in methanol (A.*R*., 99.9%) (10 ml). To the resulting clear solution was added CdCl_2_.6H_2_O (0.5 mmol, 149 mg) in methanol (10 ml). After keeping the resulting mixture in air to evaporate about half of the solvent, colourless blocks of (I) were deposited. The crystals were isolated, washed with ethanol three times (Yield 74%). Analysis: found: C, 25.98; H, 2.66; N, 6.08; S, 13.84; C_10_H_12_CdN_2_O_8_S_2_ requires: C, 25.82; H, 2.58; N, 6.02; S, 14.27.

### Refinement

The C-bound H atoms were included in the riding model approximation with C—H = 0.93–0.96 Å, and with *U*_iso_(H)= 1.2*U*_eq_(C)-1.5*U*_eq_(C). The water-bound H atoms were located in difference Fourier maps and the O—H distances were refined without constraint, see Table 1 for distances.

### Crystal data

**Table:**

[Cd(C_5_H_4_NO_3_S)_2_(H_2_O)_2_]	*F*_000_ = 460
*M**_r_* = 464.74	*D*_x_ = 2.065 Mg m^−^^3^
Monoclinic, *P*2_1_/*c*	Mo *K*α radiation λ = 0.71073 Å
Hall symbol: -P 2ybc	Cell parameters from 3022 reflections
*a* = 7.7480 (11) Å	θ = 3.1–26.3º
*b* = 13.264 (2) Å	µ = 1.78 mm^−^^1^
*c* = 7.3291 (11) Å	*T* = 294 (2) K
β = 97.081 (2)º	Block, colourless
*V* = 747.47 (19) Å^3^	0.26 × 0.22 × 0.18 mm
*Z* = 2	

### Data collection

**Table:**

Bruker SMART CCD area-detector diffractometer	1520 independent reflections
Radiation source: fine-focus sealed tube	1396 reflections with *I* > 2σ(*I*)
Monochromator: graphite	*R*_int_ = 0.023
*T* = 298(2) K	θ_max_ = 26.3º
φ and ο scans	θ_min_ = 2.7º
Absorption correction: multi-scan(SADABS; Bruker, 1998)	*h* = −9→6
*T*_min_ = 0.674, *T*_max_ = 0.740	*k* = −16→15
4111 measured reflections	*l* = −4→9

### Refinement

**Table:**

Refinement on *F*^2^	Hydrogen site location: inferred from neighbouring sites
Least-squares matrix: full	H atoms treated by a mixture of independent and constrained refinement
*R*[*F*^2^ > 2σ(*F*^2^)] = 0.021	*w* = 1/[σ^2^(*F*_o_^2^) + (0.0952*P*)^2^ + 1.5031*P*] where *P* = (*F*_o_^2^ + 2*F*_c_^2^)/3
*wR*(*F*^2^) = 0.053	(Δ/σ)_max_ = 0.001
*S* = 1.10	Δρ_max_ = 0.42 e Å^−^^3^
1520 reflections	Δρ_min_ = −0.73 e Å^−^^3^
115 parameters	Extinction correction: SHELXL, Fc^*^=kFc[1+0.001xFc^2^λ^3^/sin(2θ)]^-1/4^
Primary atom site location: structure-invariant direct methods	Extinction coefficient: 0.087 (3)
Secondary atom site location: difference Fourier map	

### Special details

**Table:**

Geometry. All e.s.d.'s (except the e.s.d. in the dihedral angle between two l.s. planes) are estimated using the full covariance matrix. The cell e.s.d.'s are taken into account individually in the estimation of e.s.d.'s in distances, angles and torsion angles; correlations between e.s.d.'s in cell parameters are only used when they are defined by crystal symmetry. An approximate (isotropic) treatment of cell e.s.d.'s is used for estimating e.s.d.'s involving l.s. planes.
Refinement. Refinement of *F*^2^ against ALL reflections. The weighted *R*-factor *wR* and goodness of fit *S* are based on *F*^2^, conventional *R*-factors *R* are based on *F*, with *F* set to zero for negative *F*^2^. The threshold expression of *F*^2^ > σ(*F*^2^) is used only for calculating *R*-factors(gt) *etc*. and is not relevant to the choice of reflections for refinement. *R*-factors based on *F*^2^ are statistically about twice as large as those based on *F*, and *R*- factors based on ALL data will be even larger.

### Fractional atomic coordinates and isotropic or equivalent isotropic displacement parameters (Å^2^)

**Table:**

	*x*	*y*	*z*	*U*_iso_*/*U*_eq_	
Cd1	0.0000	0.5000	0.5000	0.02008 (12)	
S1	0.71583 (7)	0.62259 (4)	0.77922 (8)	0.02183 (15)	
N1	0.2044 (2)	0.62074 (14)	0.6090 (3)	0.0246 (4)	
O1	0.7687 (2)	0.57880 (16)	0.6122 (3)	0.0449 (5)	
O2	0.7069 (2)	0.54880 (14)	0.9231 (3)	0.0392 (5)	
O3	0.8146 (2)	0.71095 (13)	0.8402 (3)	0.0377 (4)	
C1	0.3697 (3)	0.59388 (16)	0.6647 (3)	0.0234 (5)	
H1	0.3979	0.5257	0.6722	0.028*	
C2	0.4994 (3)	0.66450 (17)	0.7113 (3)	0.0202 (4)	
C3	0.4585 (3)	0.76576 (17)	0.7005 (4)	0.0276 (5)	
H3	0.5439	0.8143	0.7300	0.033*	
C4	0.2885 (3)	0.79362 (18)	0.6450 (4)	0.0317 (5)	
H4	0.2572	0.8613	0.6380	0.038*	
C5	0.1657 (3)	0.71933 (18)	0.6000 (3)	0.0268 (5)	
H5	0.0514	0.7385	0.5619	0.032*	
O4	0.0841 (3)	0.40325 (16)	0.7537 (3)	0.0359 (4)	
H4A	0.140 (4)	0.426 (2)	0.845 (5)	0.046 (10)*	
H4B	0.113 (4)	0.348 (3)	0.733 (5)	0.043 (9)*	

### Atomic displacement parameters (Å^2^)

**Table:**

	*U*^11^	*U*^22^	*U*^33^	*U*^12^	*U*^13^	*U*^23^
Cd1	0.01370 (15)	0.02211 (16)	0.02355 (16)	0.00007 (8)	−0.00117 (9)	−0.00150 (8)
S1	0.0152 (3)	0.0238 (3)	0.0254 (3)	−0.0008 (2)	−0.0021 (2)	−0.0035 (2)
N1	0.0181 (9)	0.0241 (9)	0.0305 (10)	0.0004 (8)	−0.0018 (8)	−0.0032 (8)
O1	0.0238 (9)	0.0694 (14)	0.0411 (11)	0.0142 (9)	0.0027 (8)	−0.0210 (10)
O2	0.0327 (10)	0.0345 (10)	0.0475 (12)	−0.0032 (8)	−0.0070 (8)	0.0145 (9)
O3	0.0270 (9)	0.0304 (9)	0.0518 (11)	−0.0079 (7)	−0.0107 (8)	−0.0012 (9)
C1	0.0191 (11)	0.0197 (10)	0.0301 (12)	0.0014 (8)	−0.0016 (9)	−0.0018 (9)
C2	0.0173 (10)	0.0245 (11)	0.0189 (10)	0.0005 (8)	0.0022 (8)	−0.0020 (8)
C3	0.0231 (11)	0.0207 (11)	0.0391 (13)	−0.0044 (9)	0.0049 (10)	−0.0049 (10)
C4	0.0297 (13)	0.0211 (11)	0.0444 (15)	0.0042 (10)	0.0047 (11)	−0.0008 (10)
C5	0.0187 (11)	0.0296 (12)	0.0313 (12)	0.0056 (9)	−0.0001 (9)	−0.0007 (10)
O4	0.0454 (11)	0.0315 (10)	0.0280 (10)	0.0043 (9)	−0.0059 (8)	0.0025 (8)

### Geometric parameters (Å, °)

**Table:**

Cd1—O4^i^	2.2872 (18)	O1—Cd1^iv^	2.3113 (17)
Cd1—O4	2.2873 (18)	C1—C2	1.386 (3)
Cd1—O1^ii^	2.3113 (17)	C1—H1	0.9300
Cd1—O1^iii^	2.3113 (17)	C2—C3	1.380 (3)
Cd1—N1^i^	2.3233 (18)	C3—C4	1.380 (4)
Cd1—N1	2.3234 (18)	C3—H3	0.9300
S1—O3	1.4404 (18)	C4—C5	1.382 (3)
S1—O2	1.4466 (19)	C4—H4	0.9300
S1—O1	1.4587 (19)	C5—H5	0.9300
S1—C2	1.779 (2)	O4—H4A	0.81 (3)
N1—C5	1.341 (3)	O4—H4B	0.79 (3)
N1—C1	1.344 (3)		
			
O4^i^—Cd1—O4	180	C5—N1—Cd1	121.09 (15)
O4^i^—Cd1—O1^ii^	83.10 (8)	C1—N1—Cd1	120.36 (15)
O4—Cd1—O1^ii^	96.90 (8)	S1—O1—Cd1^iv^	142.09 (12)
O4^i^—Cd1—O1^iii^	96.90 (8)	N1—C1—C2	122.1 (2)
O4—Cd1—O1^iii^	83.10 (8)	N1—C1—H1	119.0
O1^ii^—Cd1—O1^iii^	180	C2—C1—H1	119.0
O4^i^—Cd1—N1^i^	89.62 (7)	C3—C2—C1	119.3 (2)
O4—Cd1—N1^i^	90.38 (7)	C3—C2—S1	121.48 (17)
O1^ii^—Cd1—N1^i^	84.76 (7)	C1—C2—S1	119.21 (17)
O1^iii^—Cd1—N1^i^	95.24 (7)	C4—C3—C2	118.8 (2)
O4^i^—Cd1—N1	90.37 (7)	C4—C3—H3	120.6
O4—Cd1—N1	89.63 (7)	C2—C3—H3	120.6
O1^ii^—Cd1—N1	95.24 (7)	C3—C4—C5	118.9 (2)
O1^iii^—Cd1—N1	84.76 (7)	C3—C4—H4	120.5
N1^i^—Cd1—N1	180	C5—C4—H4	120.5
O3—S1—O2	113.30 (12)	N1—C5—C4	122.7 (2)
O3—S1—O1	113.01 (13)	N1—C5—H5	118.6
O2—S1—O1	112.70 (13)	C4—C5—H5	118.6
O3—S1—C2	106.20 (11)	Cd1—O4—H4A	122 (2)
O2—S1—C2	106.70 (11)	Cd1—O4—H4B	115 (2)
O1—S1—C2	104.03 (10)	H4A—O4—H4B	112 (3)
C5—N1—C1	118.18 (19)		
			
O4^i^—Cd1—N1—C5	42.65 (19)	N1—C1—C2—S1	−178.71 (18)
O4—Cd1—N1—C5	−137.35 (19)	O3—S1—C2—C3	8.3 (2)
O1^ii^—Cd1—N1—C5	−40.45 (19)	O2—S1—C2—C3	129.5 (2)
O1^iii^—Cd1—N1—C5	139.55 (19)	O1—S1—C2—C3	−111.2 (2)
O4^i^—Cd1—N1—C1	−130.30 (18)	O3—S1—C2—C1	−173.02 (19)
O4—Cd1—N1—C1	49.70 (18)	O2—S1—C2—C1	−51.9 (2)
O1^ii^—Cd1—N1—C1	146.60 (18)	O1—S1—C2—C1	67.5 (2)
O1^iii^—Cd1—N1—C1	−33.40 (18)	C1—C2—C3—C4	0.6 (4)
O3—S1—O1—Cd1^iv^	65.4 (2)	S1—C2—C3—C4	179.29 (19)
O2—S1—O1—Cd1^iv^	−64.7 (2)	C2—C3—C4—C5	−0.8 (4)
C2—S1—O1—Cd1^iv^	−179.9 (2)	C1—N1—C5—C4	0.2 (4)
C5—N1—C1—C2	−0.4 (3)	Cd1—N1—C5—C4	−172.90 (19)
Cd1—N1—C1—C2	172.76 (16)	C3—C4—C5—N1	0.4 (4)
N1—C1—C2—C3	0.0 (3)		

Symmetry codes: (i) −*x*, −*y*+1, −*z*+1; (ii) *x*−1, *y*, *z*; (iii) −*x*+1, −*y*+1, −*z*+1; (iv) *x*+1, *y*, *z*.

### Hydrogen-bond geometry (Å, °)

**Table:**

*D*—H···*A*	*D*—H	H···*A*	*D*···*A*	*D*—H···*A*
C5—H5···O3^v^	0.93	2.39	3.256 (3)	155
C4—H4···O2^vi^	0.93	2.55	3.422 (3)	157
O4—H4B···O3^vii^	0.79 (3)	1.99 (4)	2.780 (3)	176 (3)
O4—H4A···O2^viii^	0.81 (3)	1.98 (3)	2.773 (3)	168 (3)

Symmetry codes: (v) *x*−1, −*y*+3/2, *z*−1/2; (vi) −*x*+1, *y*+1/2, −*z*+3/2; (vii) −*x*+1, *y*−1/2, −*z*+3/2; (viii) −*x*+1, −*y*+1, −*z*+2.
